# Evaluation of endogenous reference genes in *Bactrocera cucurbitae* by qPCR under different conditions

**DOI:** 10.1371/journal.pone.0202829

**Published:** 2018-12-17

**Authors:** Yanan Zhang, Zhi Gong, Lei Li, Liming Niu, Yueguan Fu

**Affiliations:** 1 Environment and Plant Protection Institute, Chinese Academy of Tropical Agricultural Sciences, Haikou, Hainan, China; 2 College of Tropical Agriculture and Forestry, Hainan University, Haikou, Hainan, China; East Carolina University, UNITED STATES

## Abstract

*Bactrocera cucurbitae* (melon flies) are prominent invasive pests in southern China. To screen for a stable reference gene in melon flies suitable for comparing tissue samples subjected to different conditions in four categories (temperature, insect stage, days of age and gender), the expression of 12 candidate reference genes under different treatment conditions was analyzed by real-time fluorescent quantitative PCR. The results obtained from a comprehensive analysis with geNorm, NormFinder, BestKeeper and RefFinder software showed that the most stable reference gene was RPL60, and the least stable reference gene was actin-5. We used a heat shock protein gene (HSP-90) to verify the results, and the conclusion was consistent. When the reference gene RPL60 was used as the reference gene, the relative expression of HSP-90 was essentially constant with the prolongation of treatment time. When actin-5 was used, HSP-90 expression changed markedly with treatment time. The results of this study can be used for further research on gene expression in*Bactrocera cucurbitae*.

## Introduction

*Bactrocera cucurbitae* (Diptera, Tephritidae), or melon flies, are important invasive pests widely distributed in temperate, tropical, and sub-tropical regions of the world[1–2]. Melon flies damage a variety of fruits and vegetables[3], including bitter gourd, cucumber, pumpkin, muskmelon, watermelon, and papaya[4]; and they have caused huge economic losses in the south of China[5]. Melon flies are world quarantine pests[6–8]; with the increasing globalization of trade, melon flies are becoming of more quarantine concern than ever before. It is a long-term and arduous task to study the development, occurrence, damage, genetic differentiation and control of melon flies. Molecular technology has begun to be widely used in studying the melon fly. With the determination of melon fly transcriptome data, we can explore the expression of different genes in these insects, such as differences in the expression of odorant binding proteins, the expression of genes due to stress. Gene expression data, which can be obtained by quantitative real-time PCR, can also help explain the behavioral mechanisms of melon flies.

Quantitative real-time PCR (qRT-PCR) is an established technique for quantifying mRNA in biological samples[9]. It has been widely used in gene expression analysis because it is sensitive, accurate, reproducible and quantitative[10–14]. However, qRT-PCR still has some limitations in gene expression analysis, data analysis and subsequent interpretation due to variations in transcription and amplification efficiencies among different samples[12, 15–17]. Thus, when qRT-PCR is performed, it is necessary to normalize gene expression analysis data by measuring expression of reliable reference genes in the same samples in parallel[10–20]. To make sure expression of a candidate reference gene occurs at a constant level, each candidate reference gene should be evaluated under specific experimental conditions [15].

In recent years, there have been several studies on reference genes in insects such as *Bactrocera dorsalis*[21], *Drosophila melanogaster*[22], *Bactrocera minax*[23–24], and *Anastrepha oblique*[25]. Previously, reference genes in *Bactrocera cucurbitae* have seldom been reported. We evaluated the stability of 12 reference genes—a tubulin gene (β-tubulin); actin genes (β-actin, actin-5); ribosomal protein genes (RPL60, RPL40); extension factor genes (EF1-α, EF1-β); succinate dehydrogenase genes (SD-1,SD-2); ribosomal RNA genes (5S rRNA, 28S rRNA)[26]; and a ubiquitin-like protein gene (UBQ-5)—at different developmental stages and under different temperature conditions. The aim of this study was to identify appropriate reference genes for studying melon fly gene expression profiles under different experimental conditions.

## 1 Materials and methods

### 1.1 Insects

Melon fly populations were reared in an environmental chamber at 25±1°C with a 14:10 (light:dark) photoperiod and 65–75% relative humidity. To make the results more accurate, the ages of the melon fly larvae were synchronized before the experiment.

### 1.2 Candidate reference genes

Selected candidate reference genes included genes for tubulin (β-tubulin); actin (β-actin, actin-5), ribosomal RNA (5S rRNA, 28S rRNA); ribosomal proteins (RPL60, RPL40); extension factors (EF1-α, EF1-β); succinate dehydrogenase (SD-1, SD-2); and a ubiquitin-like protein (UBQ-5). The melon fly transcriptome was screened for the sequence information of candidate reference genes, and we used a heat shock protein gene, HSP-90, as a validation gene. Genious 10.0 was used to design primers for real-time PCR analysis. The PCR primer sequences used for quantitative expression are shown in Table 1.

**Table 1:** Primer sequences used in real-time PCR of *Bactrocera cucurbitae* reference genes.

Name	Description	Gene ID	Forward (F)and reverse (R)primer	Tm	Product Size
β-Tubulin	Tubulin beta-2 chain	CL2041.Contig3_All	F: 5’-ATTTGGTCAGTCAGGAGCGG-3’	60°C	124bp
			R: 5’-TCCCTGTAGGCAATCGCATC-3’	59.9°C	
Actin-5	indirect flight muscle	Unigene8193_All	F: 5’- CGGAATGCTTTAGCGCAGTT-3’	59.6°C	110bp
			R: 5’- GCCTTCAGCATGATGTACCG-3’	59.1°C	
β-Actin	indirect flight muscle	Unigene10544_All	F: 5’- ATTGCGGAATGCTTTAGCGC-3’	60.2°C	105bp
			R: 5’- ATGATGTACCGCTGGCAGTC -3’	60.2°C	
28S rRNA	28S ribosome RNA	Unigene10359_All	F: 5’- GCCACAAGCCAGTTATCCCT-3’	60°C	93bp
			R: 5’- ACAGCAAAAGCTCGGCCTAT-3’	60°C	
5S rRNA	5S ribosome RNA	Unigene9024_All	F: 5’- AACGACCATACCACGCTGAA -3’	59.7°C	92bp
			R: 5’- AGCGGTCCCCCATCTAAGTA-3’	59.7°C	
RPL60	60S ribosomal protein L32	Unigene10046_All	F: 5’- CGCACAAATGGCGTAAACCA-3’	60°C	119bp
			R: 5’- TGGTAGCATATGACGGGTGC -3’	59.9°C	
RPL40	40S ribosomal protein S20	Unigene7001_All	F: 5’- CAAGGCGCTGATTCTGCATC -3’	60°C	121bp
			R: 5’- CGCGCAGATTCTGGTTCTTG-3’	59.9°C	
EF1-α	Elongation factor 1-α	CL3926.Contig3_All	F: 5’- CCAAGCCTTTGTGCGTTGAA -3’	59.9°C	124bp
			R: 5’- CCGGAGGCATCCTTGAAGTT -3’	60°C	
EF1-β	Elongation factor 1-β	Unigene10513_All	F: 5’-AAGAAACCCGCGCTTATTGC -3’	59.8°C	136bp
			R: 5’-ATTTGGAGGCACCCCACAAA -3’	60.1°C	
SD-1	Succinate Dehydrogenase	CL4176.Contig1_All	F: 5’- GGTGAACGCAAAGGGTGAAC-3’	60°C	96bp
			R: 5’- TCGATGGTCATTGAACGCGA -3’	60.1°C	
SD-2	Succinate Dehydrogenase	Unigene5742_All	F: 5’-TGCGGCTCATGTGCTATGAA -3’	60.1°C	96bp
			R: 5’- GGGGTACACTTTAAGCGGCT-3’	60°C	
UBQ-5	Ubiquitin-like protein 5	Unigene11936_All	F: 5’- AGTTGATTGCCGCTCAGACT-3’	59.7°C	123bp
			R: 5’- GCTCCAAATTCATGCCATCGT -3’	59.6°C	
HSP-90	Heat Shock Protein 90	Unigene160_All	F: 5’-GATATGCCTCAGCGGTCTCC-3	60°C	134bp
			R: 5’-GCCATTCAAAGTGGTGAGCG-3’	60°C	

### 1.3 Treatment conditions

Mature larvae(7-day larvae), 4-day pupae, and 1-, 7-, 15-, and 30-day female and male adults were sampled to test the expression of candidate reference genes. These flies were raised at 25°C. Mature larvae, 4-day pupae, and 7-day female and male adults were the control groups. The two treatments were as follows:

For the high temperature treatment, whole developmental stages of melon flies were carried out in a 33°C artificial climatic incubator. Mature larvae, 4-day pupae, and 7-day adults were sampled during development.

The low temperature treatment was similar to the high temperature treatment, except that the treatment temperature was 19°C.

### 1.4 RNA extraction and reverse transcription

Total RNA was extracted using a TransZol Up Plus RNA Kit (Beijing TransGen Biotech Co., Ltd) following the manufacturer’s instructions. RNA sample purity was analyzed by means of the A260/A280 absorbance ratio with a Nanodrop ND-1000 (Thermo Scientific), and RNA integrity was confirmed using 1.0% agarose gel electrophoresis to test for a specific expected band. RNA was treated with DNase I (GIBCO-BRL, Gaithersburg, MD), and cDNA template was synthesized from 5 μg of total RNA using a First-Strand cDNA Synthesis Kit (Thermo Fisher Scientific Inc) with 1 μl of oligo (dT) as the primer (10 μM).

### 1.5 Quantitative real-time PCR

The qRT-PCR reactions were performed on a QuantStudio 6 Flex system (Thermo Fisher Scientific Inc.) according to the manufacturer’s instructions for TransStart Tip Green SuperMix (Beijing TransGen Biotech Co., Ltd.). Reaction volumes were 20 µl and included 1 μl of cDNA, 0.4 μl of each primer (10 μM), 10 μl of 2× TransStart Tip Green qPCR SuperMix, 0.4 μl of passive reference dye (50×), and 8 μl of ddH_2_O. The thermal cycling conditions were as follows: step 1, 95°C for 3 min; step 2, 40 cycles of 95°C for 5 sec and 60°C for 30 sec; and step 3, 95°C for 15 sec, 60°C for 1 min, and 95°C for 15 sec. Information was collected at stage 2 of step 2. A standard curve was created with a 4-fold dilution series of cDNA, and PCR efficiency (E) values between 94% and 119% were determined for all primer pairs, with typical correlation coefficients (R^2^) greater than or equal to 0.98.

To evaluate the stability of melon fly reference genes under different treatments, the expression of three target genes under varying durations of heat stress was analyzed by qRT-PCR. The most stable gene, RPL60, and the gene with the lowest stability, actin-5, were used as reference genes to analyze the expression stability of the heat shock protein.

### 1.6 Statistical methods

Statistical analyses were conducted using the SPSS package (version 19). Prior to all statistical analyses, data were examined for violations of normality assumptions using the Kolmogorov-Smirnov test. The effects of different development stages and different stress conditions on reference gene expression were analyzed by the least significant difference (LSD) test after one-way ANOVA[27]. The results were expressed as the means ± standard errors (mean ± SEM). The differences were considered significant when P values were ≤ 0.05.

### 1.7 Expression stability analysis of candidate reference genes

Expression stability of the candidate reference genes was analyzed using the delta Ct method, BestKeeper[28], geNorm version 3.5 [29], NormFinder version 0.953[30], and RefFinder (http://150.216.56.64/referencegene.php). All the analysis procedures followed the manuals of each method.

### 1.8 Validation of the recommended reference genes

To validate the stability of the identified reference genes, the expression levels of two target genes were analyzed under different experimental conditions. We used the heat shock protein gene HSP-90 to validate the expression levels of the most stable and least stable reference genes for melon fly during high-temperature (33°C) conditions. Adults were sampled after 2, 4, 6 and 8 hours of heat treatment. RNA was extracted, and cDNA was reverse transcribed for each sample. After fluorescence quantitative PCR experiments, relative quantification of these three genes in different samples was performed using the 2^(-ΔΔCT)^ method.

## 2 Results

### 2.1 Total RNA quality testing

**Fig 1:**
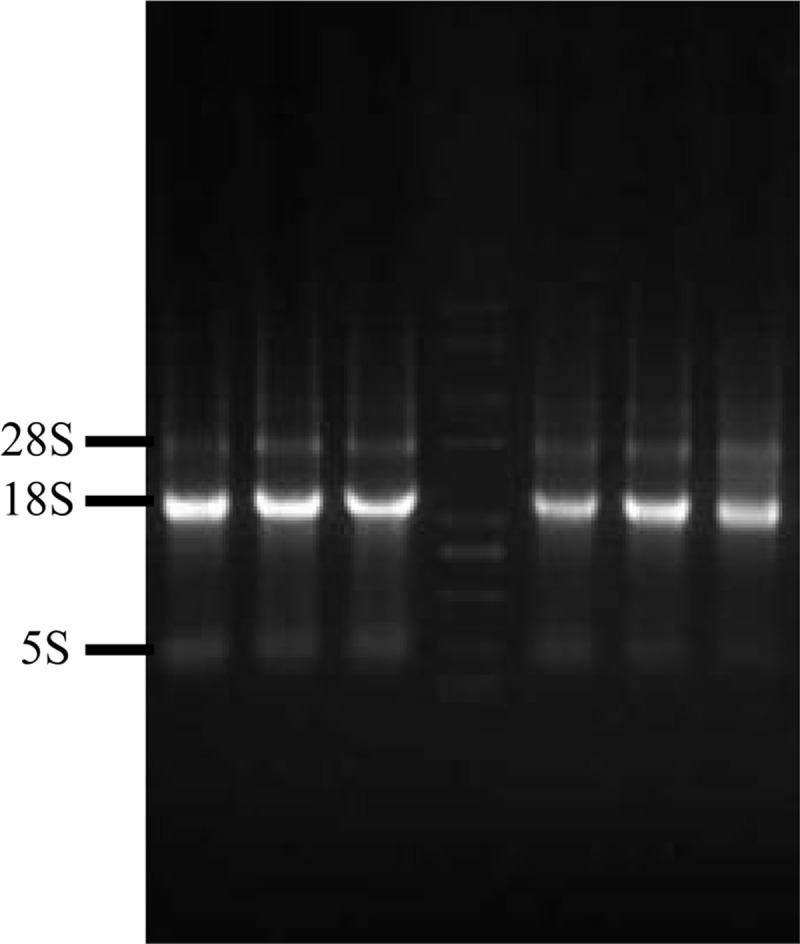
The electrophoresis results of total RNA.

Total RNA was extracted from 20 melon fly samples. The concentration and quality of the RNA were determined with a micro-UV spectrophotometer and electrophoresis. The results showed that the A260/280 ratios of all samples were between 1.8 and 2.0, and the bands were clear (Fig 1).

### 2.2 Primer optimization and qRT-PCR amplification efficiency

An RT-qPCR assay based on SYBR Green dye detection was carried out to examine the stability of the expression of the 12 candidate genes. Each sample was measured in triplicate within each run, and three independent technical replicates were performed for each experiment. A single peak in each melting curve in the RT-qPCR experiments indicated good specificity of the primers[31]. The RT-qPCR amplification efficiency (E) and correlation coefficient (R^2^) were calculated based on the slope of the calibration curves (Table 2). For the twelve candidate reference genes and three target genes, the E values ranged from 94% to 108%, between 90–110%. Correlation coefficient(R^2^) ranged from 0.9844 to 0.9988 which all above 0.98. The results showed that all 12 pairs of primers met the requirements of qRT-PCR experiments.

**Table 2:** Amplification characteristics of the twelve candidate reference genes in qRT-PCR.

Gene name	Primer sequence (F/R)	Amplicon length (bp)	Tm (°C)	R^2^	Efficiecy(%)
β-Tubulin	5’-ATTTGGTCAGTCAGGAGCGG-3’	124 bp	60	0.9962	105
	5’-TCCCTGTAGGCAATCGCATC-3’		59.9		
Actin-5	5’- CGGAATGCTTTAGCGCAGTT-3’	110 bp	59.6	0.9979	110
	5’- GCCTTCAGCATGATGTACCG-3’		59.1		
β-Actin	5’- ATTGCGGAATGCTTTAGCGC-3’	105bp	60.2	0.9971	107
	5’-ATGATGTACCGCTGGCAGTC-3’		60.2		
28s rRNA	5’- GCCACAAGCCAGTTATCCCT-3’	93 bp	60	0.9953	108
	5’- ACAGCAAAAGCTCGGCCTAT-3’		60		
5S rRNA	5’- AACGACCATACCACGCTGAA -3’	92 bp	59.7	0.9938	107
	5’- AGCGGTCCCCCATCTAAGTA-3’		59.7		
RPL60	5’- CGCACAAATGGCGTAAACCA-3’	119 bp	60	0.9874	95
	5’- TGGTAGCATATGACGGGTGC -3’		59.9		
RPL40	5’- CAAGGCGCTGATTCTGCATC -3’	121 bp	60	0.9844	94
	5’- CGCGCAGATTCTGGTTCTTG-3’		59.9		
EF1-α	5’- CCAAGCCTTTGTGCGTTGAA -3’	124 bp	59.9	0.9973	103
	5’- CCGGAGGCATCCTTGAAGTT -3’		60		
EF1-β	5’-AAGAAACCCGCGCTTATTGC -3’	136 bp	59.8	0.9977	105
	5’-ATTTGGAGGCACCCCACAAA -3’		60.1		
SD-1	5’- GGTGAACGCAAAGGGTGAAC-3’	96 bp	60	0.9922	106
	5’- TCGATGGTCATTGAACGCGA -3’		60.1		
SD-2	5’-TGCGGCTCATGTGCTATGAA -3’	96 bp	60.1	0.9988	106
	5’- GGGGTACACTTTAAGCGGCT-3’		60		
UBQ-5	5’- AGTTGATTGCCGCTCAGACT-3’	123 bp	59.7	0.9927	107
	5’- GCTCCAAATTCATGCCATCGT -3’		59.6		

### 2.3 Reference gene RT-PCR analysis

**Fig 2:**
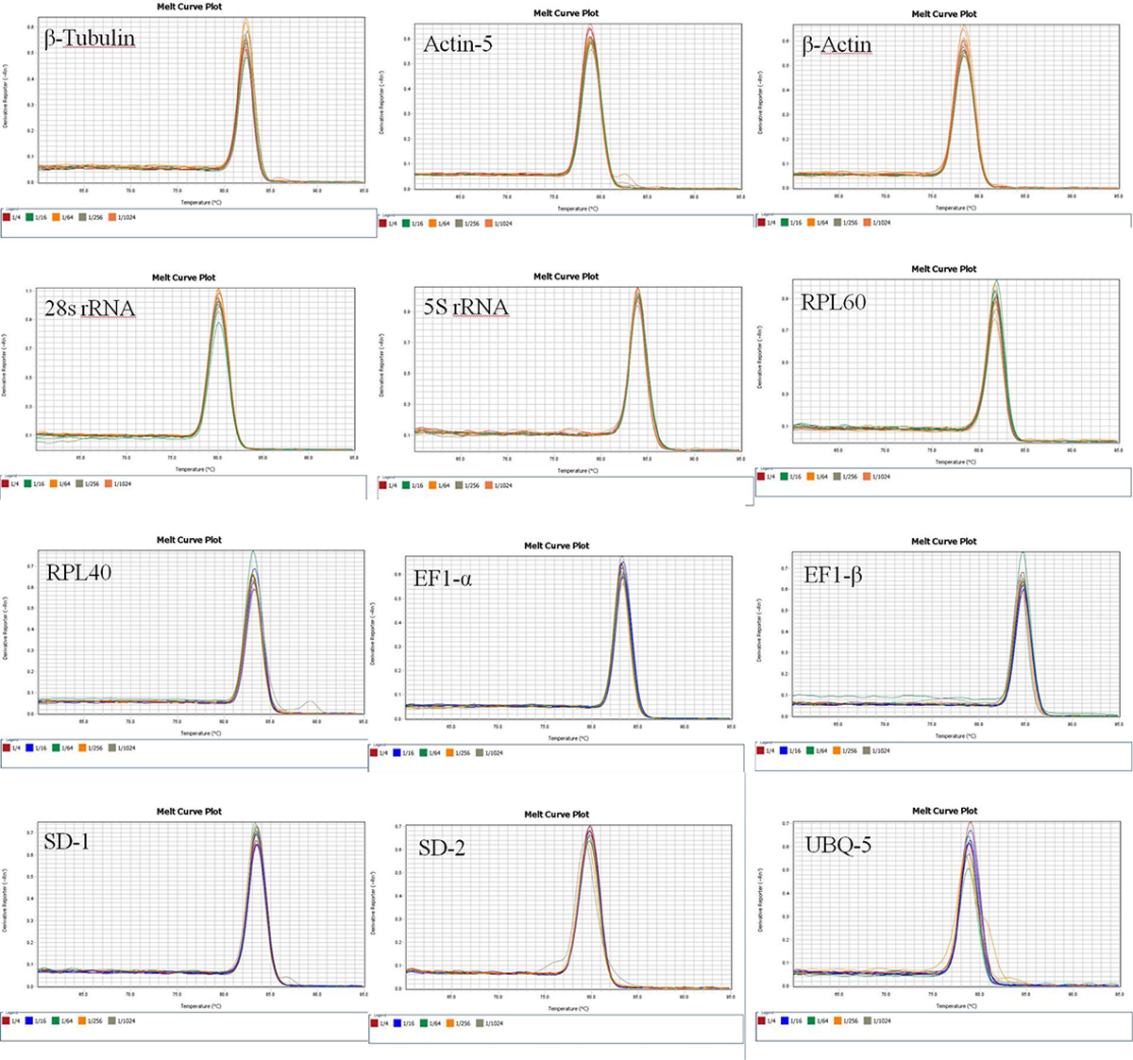
Melting curves of 12 candidate reference genes for qRT-PCR amplification.

Fluorescent quantitative PCR analysis of the melon fly cDNA template showed that there was only a single main peak in each of the melting curves for the 12 candidate internal reference genes under different experimental conditions (Fig 2); no other miscellaneous peaks appeared, and the reproducibility of the amplification curve between replicates was high.

### 2.4 Reference gene expression analysis

The stability of reference gene Ct values in different samples is an important criterion for selection. Through data analysis, we found that there were differences in the expression of each reference gene in different samples. The smaller the Ct value of the gene is, the higher the gene expression, and vice versa. The average Ct value of candidate reference genes was 12.97–30.03 in all melon fly samples. The average Ct value of RPL60, which was 17.02, was the lowest. Compared with other reference genes, RPL60 had the highest expression in all treatments. The average Ct value of SD-1 was the highest (25.75), reflecting the lowest expression of all reference genes. The standard deviations of the Ct values for 5S rRNA, EF1-β and RPL60 were relatively small, and the expression was relatively stable. Actin-5 had the largest standard deviation of Ct values and the poorest expression stability.

### 2.5 Evaluation of reference gene expression stability

#### 2.5.1 GeNorm

**Fig 3:**
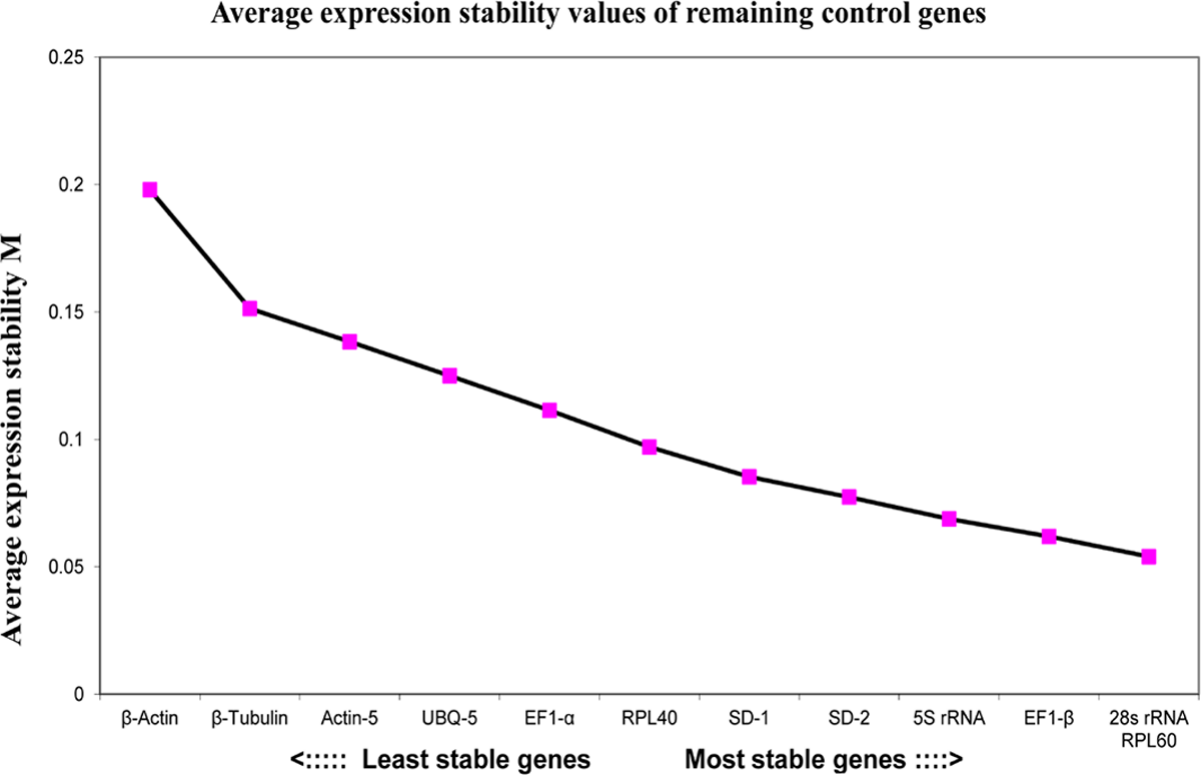
Expression stability of 12 candidate reference genes calculated by geNorm.

GeNorm analyzes the expression stability value (M) of reference genes in different samples and sorts them to determine the most stable internal control gene. The smaller the value of M is, the higher the stability. If M <1.5, the internal reference gene expression is stable. In this study, geNorm analysis showed that the M values of all 12 reference genes were less than 1.5, indicating that these genes were stable. As seen from Table 3, Figs 3 and S1, the 28S rRNA, RPL60 and EF1-β genes were most stably expressed in different samples, with M values of 0.150, 0.152 and 0.144, respectively, while β-actin was the least stably expressed, with an M value of 0.431.

**Table 3:** Expression stability of 12 candidate reference genes analyzed by GeNorm, NormFinder and BestKeeper.

Rank	GeNorm		NormFinder		BestKeeper		
	Gene	M	Gene	Stability value	Gene	SD	CV
1	EF1-β	0.144	RPL60	0.066	5S rRNA	1.18	5.54
2	5S rRNA	0.148	SD-2	0.07	EF1-β	1.27	6.96
3	28s rRNA	0.150	SD-1	0.08	RPL60	1.79	10.53
4	RPL60	0.152	EF1-β	0.091	β-Tubulin	2.67	11.02
5	SD-1	0.152	RPL40	0.093	RPL40	2.30	13.09
6	SD-2	0.160	5S rRNA	0.116	UBQ-5	2.81	13.17
7	RPL40	0.185	28s rRNA	0.139	SD-2	2.93	14.19
8	UBQ-5	0.207	EF1-α	0.142	SD-1	3.89	15.08
9	EF1-α	0.210	β-Tubulin	0.149	28s rRNA	3.16	18.36
10	Actin-5	0.215	UBQ-5	0.172	EF1-α	3.62	20.33
11	β-Tubulin	0.221	β-Actin	0.227	β-Actin	5.69	25.43
12	β-Actin	0.431	Actin-5	0.256	Actin-5	5.79	26.52

#### 2.5.2 NormFinder

**Fig 4:**
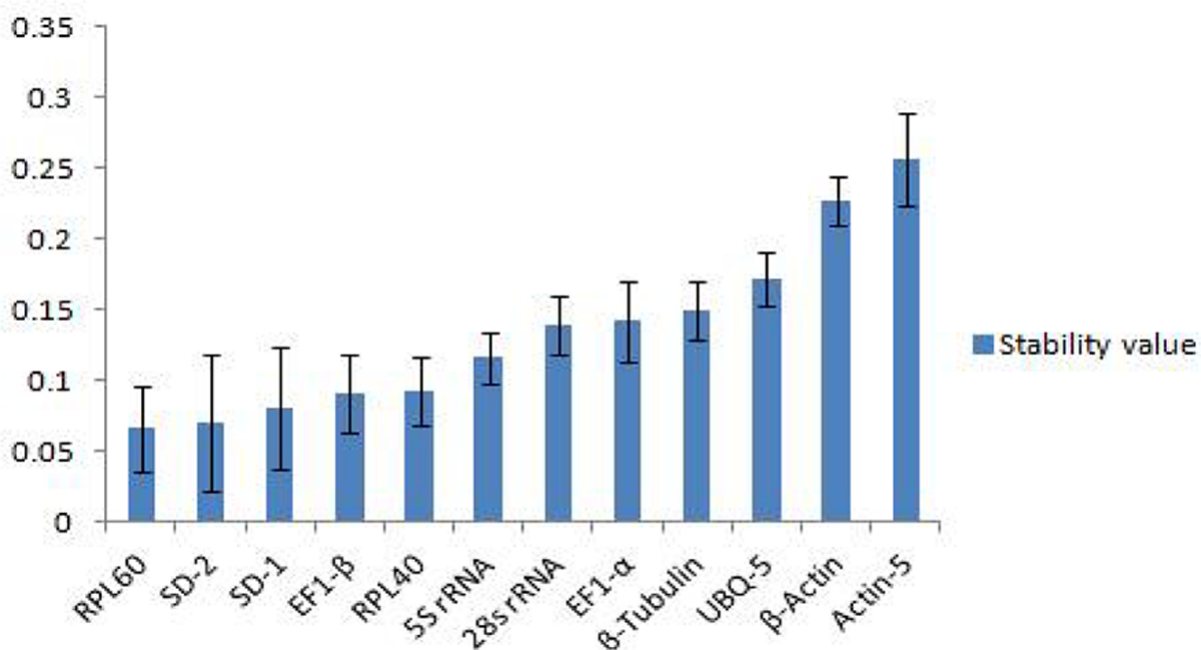
Expression stability of 12 candidate reference genes analyzed by Norm Finder (M).

The NormFinder software evaluates the stability of the candidate reference gene by combining the variance within the group and the variance between groups. A lower stability value indicates a higher stability in the expression of the reference gene[32]. We used NormFinder to further confirm the results obtained with the geNorm program. The stability ranking of the reference genes by NormFinder was slightly different from the one determined by geNorm. As shown in Table 3 and Fig 4, under the different treatments, the most stable reference gene was RPL60, followed by SD-2, SD-1, and EF1-β. The most unstable reference gene was actin-5.

#### 2.5.3 BestKeeper

BestKeeper screens for the most stable reference genes by calculating the standard deviation (SD) and coefficient of variation (CV), where a smaller standard deviation and coefficient of variation indicate better stability. As shown in Table 3 and S2 Fig, under the different treatments, the most stable reference gene was 5S rRNA, followed by EF1-β, RPL60, and β-tubulin. The most unstable reference gene was actin-5.

#### 2.5.4 RefFinder

**Fig 5:**
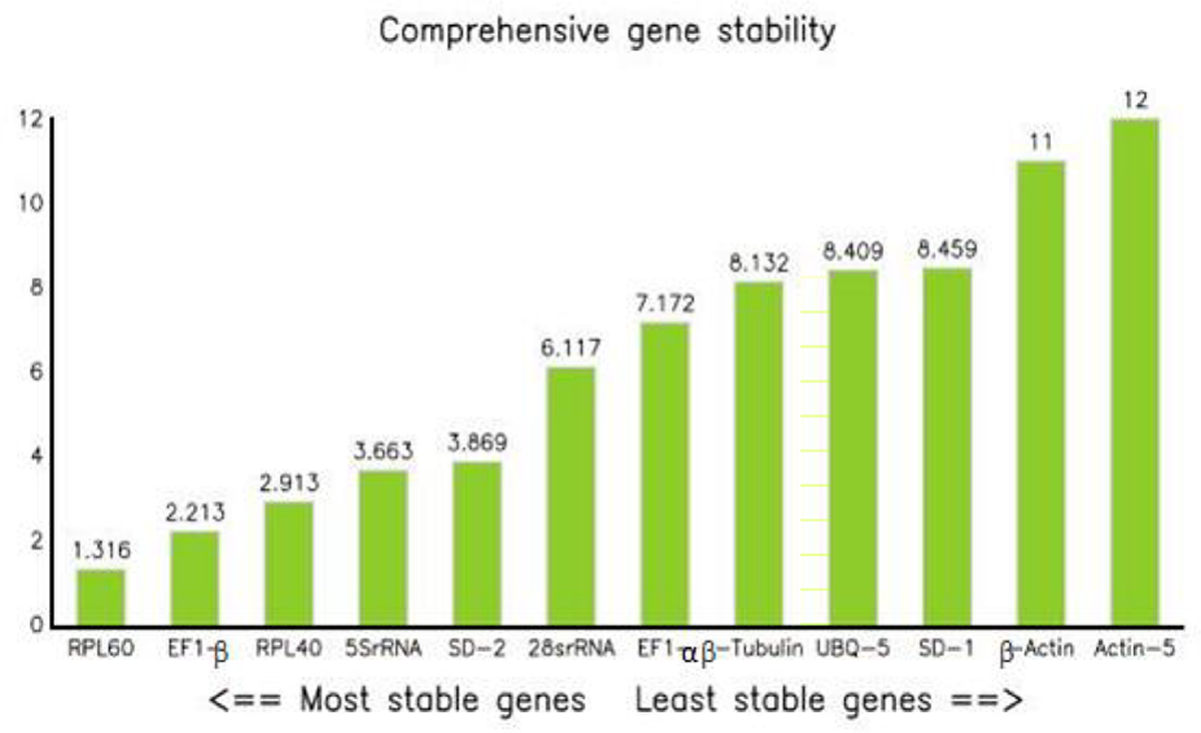
Expression stability of 12 candidate reference genes calculated by RefFinder.

RefFinder integrates the currently available major computational programs (geNorm, NormFinder, BestKeeper, and the comparative Ct method) to compare and rank the tested candidate reference genes[33]. Based on the rankings from each program, it assigns an appropriate weight to an individual gene and calculates the geometric mean of the weights for the overall final ranking. The smaller the index is, the more stable the expression of the reference gene. Table 4 and Fig 5 show that the most stable reference gene was RPL60, and the most unstable reference gene was actin-5.

**Table 4:** RefFinder ranking of the 12 candidate reference genes with respect to their expression stability under different experimental conditions.

Genes	Geomean of ranking values	Rank
RPL60	1.32	1
EF1-β	2.21	2
RPL40	2.91	3
5SrRNA	3.66	4
SD-2	3.87	5
28srRNA	6.12	6
EF1-α	7.17	7
β-Tubulin	8.13	8
UBQ-5	8.41	9
SD-1	8.46	10
β-Actin	11.00	11
Actin-5	12.00	12

### 2.6 Reference gene stability verification

**Fig 6:**
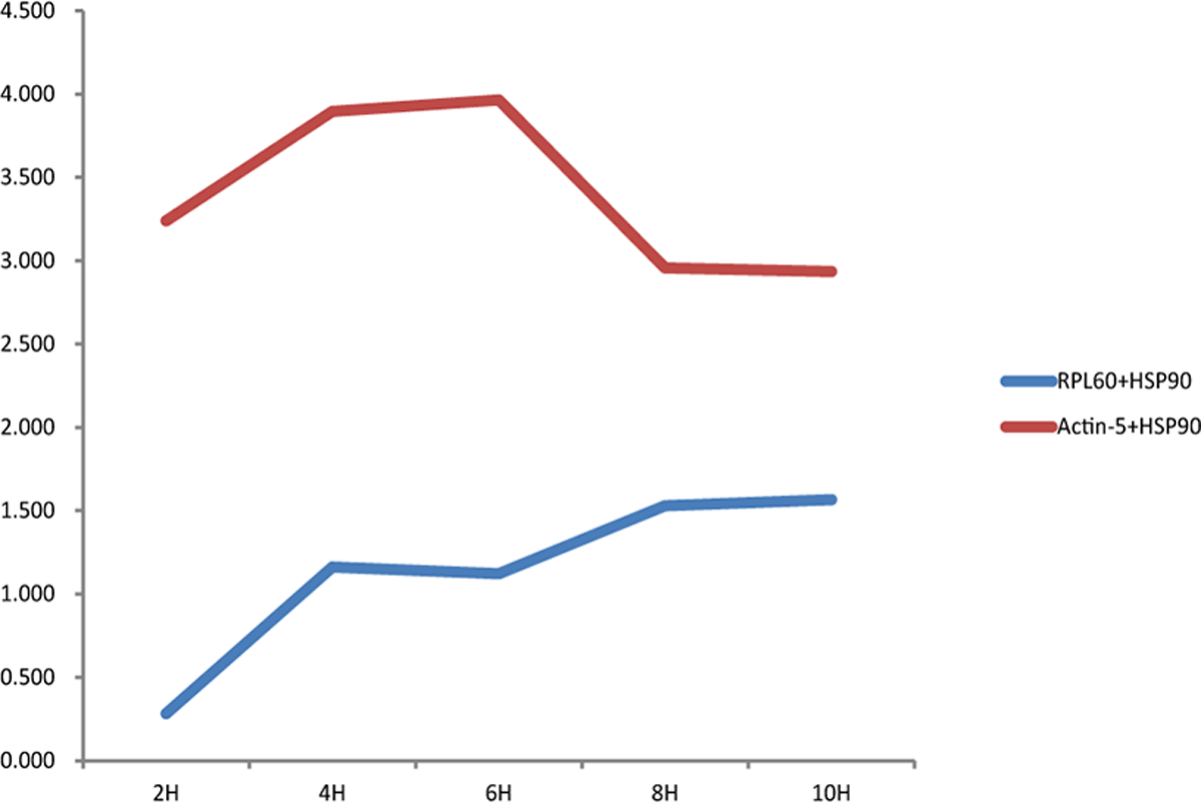
Expression of RPL60 and actin-5 under high-temperature treatment. Note: X-axis: time; Y-axis: relative quantification.

We analyzed the stability in expression of a heat shock protein using the most stable candidate reference gene, RPL60, and least stable candidate reference gene, actin-5, as internal reference genes. As seen from Fig 6, when RPL60 was used as a reference gene, the relative expression level of the target gene was consistent with the prolongation of treatment time, indicating that the gene expression was very stable. When actin-5 was used as an internal control gene, the target gene relative expression changed significantly with treatment time; thus, the expression stability was poor, and the relative expression of the target gene could not be accurately calculated.

## 3 Discussion

A stable reference gene is a prerequisite for improving the reliability of RT-qPCR. The selection of early reference genes was mainly based on their function; actin and TUB genes are the basic components of organelle skeletons, while RPL, EF and UBQ are involved in the basic biological and metabolic processes of organisms, suggesting that these genes should be stably expressed in all cellular and physiological states. The choice of the correct reference gene depends largely on the tissue being studied, the conditions of the experiment, the stages of growth and development, and the biotic or abiotic stresses and hormones present, all of which can affect the specific expression of the reference gene. Due to the influence of the internal and external environment, an ideal reference gene among all different tissues may not exist [34–35]. The expression of the ideal reference gene in different tissues, cells and different experimental conditions is only relatively stable[36]. It is also an extremely important step to select the appropriate reference gene.

We used the most commonly referenced stability evaluation software: geNorm, NormFinder and BestKeeper. NormFinder takes all candidate genes into account and ranks the candidate genes with minimal estimated intra- and inter-group variation. In contrast, geNorm sequentially excludes the least stable genes, ending up with two genes, and ranks genes by the degree of similarity of expression profile[18]. BestKeeper screens for the most stable reference genes by calculating the standard deviation and coefficient of variation[37]. The three kinds of software use different algorithms, but their results were essentially the same. Then, we used RefFinder to integrate software packages like geNorm, NormFinder, and BestKeeper to produce a final composite ranking result that would make our results more robust.

The appropriate reference gene must be selected according to the different test subjects, experimental purposes and experimental conditions. In this study, cDNA was reverse transcribed from RNA extracted from melon flies of different developmental stages (larvae, pupae and adult), different sexes (female, male), and different days of age (1-, 7-, 15-, 30 day), as well as those reared in different temperatures (31°C, 25°C, and 19°C). Twelve pairs of primers were designed for real-time quantitative PCR. The most suitable internal control gene was RPL60, which was stable under a variety of experimental conditions. Apart from the genes discussed above, another actin gene (β-actin), ribosomal RNA genes (5S rRNA, 28S rRNA) and extension factor genes (EF1-α, EF1-β), which are widely used in many research fields as reference genes, were not suitable reference genes in the melon fly. Actin as a reference gene was susceptible to treatment differences, resulting in greater error in the test results. Our study also showed that using rRNA (5S rRNA, 28S rRNA) as a single normalizer can overestimate the expression of the target gene, leading to false positive results.

With the successful sequencing of the melon fly transcriptome, real-time quantitative PCR will be more widely applicable to the functional study of melon fruit fly genes. A stable reference gene is a prerequisite for improving the reliability of RT-qPCR results. This study therefore lays the foundation for the expression analysis of functional genes in melon flies.

## Supporting information

S1 Fig Expression stability of 12 candidate reference genes calculated by geNorm. (TIF)

S2 Fig Expression stability of 12 candidate reference genes calculated by BestKeeper. (TIF)
