# Supplementary figures and images for: Evaluation of endogenous reference genes in Bactrocera cucurbitae by qPCR under different conditions
Source: PLoS One. 2018 Dec 17;13(12):e0202829. doi: 10.1371/journal.pone.0202829 (PMC6296707; doi:10.1371/journal.pone.0202829)

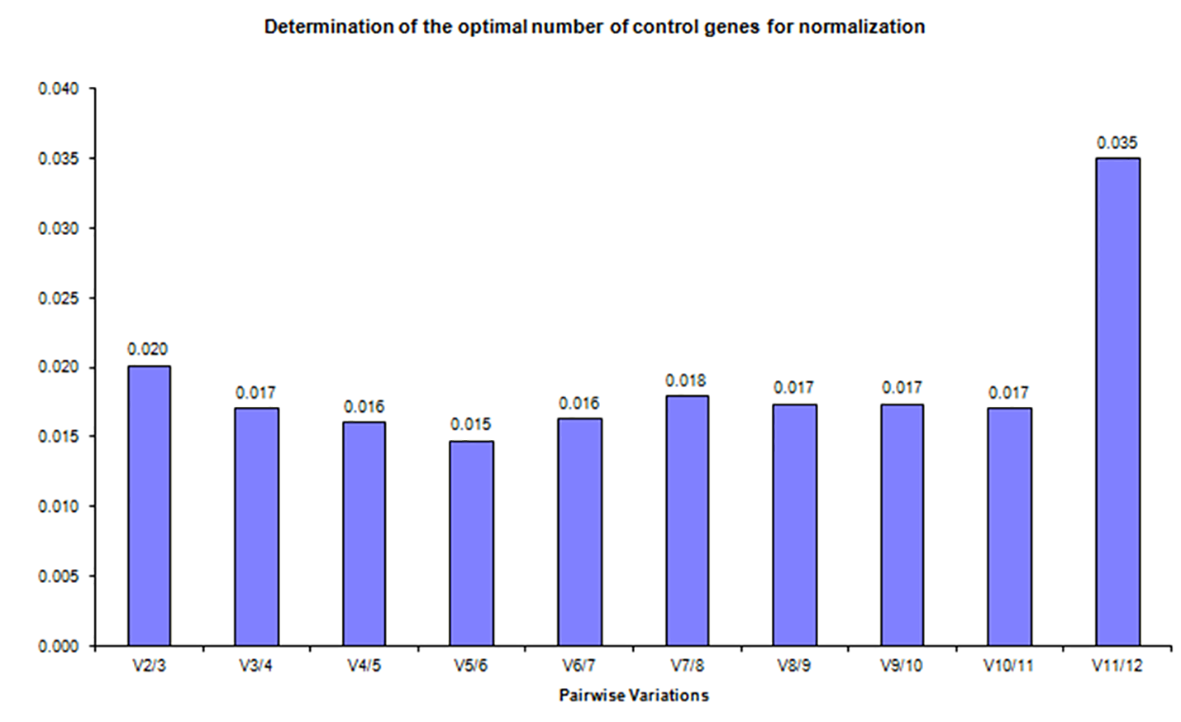

Supplement: S1 Fig — (TIF) [file pone.0202829.s001.tif]

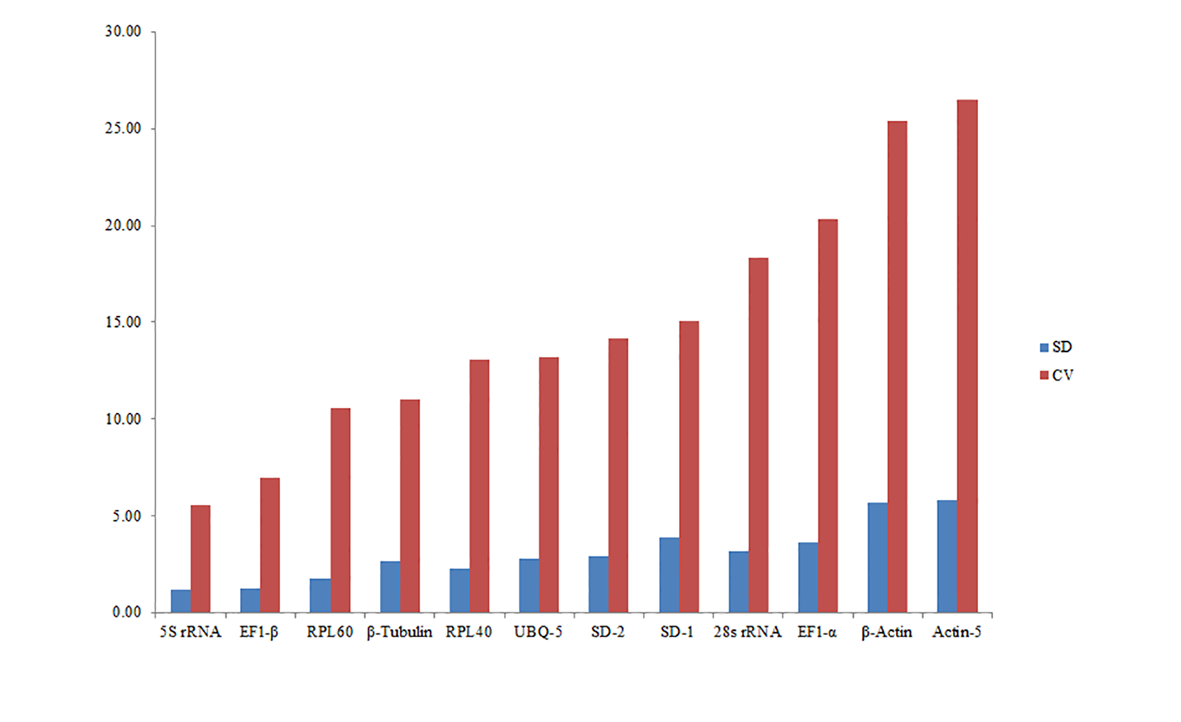

Supplement: S2 Fig — (TIF) [file pone.0202829.s002.tif]
